# HEART FAILURE AND SIT-TO-STAND SCORE ON LOWER BODY MOBILITY DISABILITY: NATIVITY DIFFERENCES

**DOI:** 10.1093/geroni/igae098.3015

**Published:** 2024-12-31

**Authors:** Melanie Navos, Soham Al Snih

**Affiliations:** The University of Texas Medical Branch, Baytown, Texas, United States; University of Texas Medical Branch in Galveston, Galveston, Texas, United States

## Abstract

We examined nativity differences in the relationship of heart failure and five-times sit-to-stand scores with lower body mobility disability over 12-years of follow-up among Mexican-American aged ≥ 75 years who were non-disabled at baseline. Participants (N=987) were from the Hispanic Established Population for the Epidemiologic Study of the Elderly (2004/05-2016). Measures included socio-demographics, heart failure (HF), multimorbidity, falls, depressive symptoms, cognitive function, handgrip strength, sit-to-stand (STS) test, and lower body disability (limitations in ≥ 1 of transferring, bathing, toileting, and walking across the room). Participants were grouped into four groups: No HF-high STS (n=402), no HF-low STS (n=381), HF-low STS (99), HF-high STS (n=105). We used the generalized estimating equation models to estimate the odds ratio (OR) and 95% confidence interval (CI) of lower body disability as a function of HF and STS groups. Compared to those with No HF-high STS group, foreign-born in the HF and high STS group had lower odds (OR=0.48, 95% CI=0.24-.95) of reporting mobility disability over time after controlling for all covariates. Foreign-born and US-born in the groups of HF and low STS had greater odds (OR=2.31, 95% CI=1.14-4.67 and 7.03, 95% CI=3.57-13.81) of reporting mobility disability over time after controlling for all covariates. In conclusion, Mexican-American older adults with HF who performed high in five-times STS were at lower risk for lower body disability and the risk differed by nativity. Interventions to improve strength in lower extremities in those with HF may prevent or reduce disability in this population

